# HIV-1 latency in actively dividing human T cell lines

**DOI:** 10.1186/1742-4690-5-37

**Published:** 2008-04-25

**Authors:** Rienk E Jeeninga, Ellen M Westerhout, Marja L van Gerven, Ben Berkhout

**Affiliations:** 1 Laboratory of Experimental Virology, Department of Medical Microbiology, Center for Infection and Immunity Amsterdam (CINIMA), Academic Medical Center, University of Amsterdam, The Netherlands

## Abstract

**Background:**

Eradication of HIV-1 from an infected individual cannot be achieved by current drug regimens. Viral reservoirs established early during the infection remain unaffected by anti-retroviral therapy and are able to replenish systemic infection upon interruption of the treatment. Therapeutic targeting of viral latency will require a better understanding of the basic mechanisms underlying the establishment and long-term maintenance of HIV-1 in resting memory CD4 T cells, the most prominent reservoir of transcriptional silent provirus. However, the molecular mechanisms that permit long-term transcriptional control of proviral gene expression in these cells are still not well understood. Exploring the molecular details of viral latency will provide new insights for eventual future therapeutics that aim at viral eradication.

**Results:**

We set out to develop a new in vitro HIV-1 latency model system using the doxycycline (dox)-inducible HIV-rtTA variant. Stable cell clones were generated with a silent HIV-1 provirus, which can subsequently be activated by dox-addition. Surprisingly, only a minority of the cells was able to induce viral gene expression and a spreading infection, eventhough these experiments were performed with the actively dividing SupT1 T cell line. These latent proviruses are responsive to TNFα treatment and alteration of the DNA methylation status with 5-Azacytidine or genistein, but not responsive to the regular T cell activators PMA and IL2. Follow-up experiments in several T cell lines and with wild-type HIV-1 support these findings.

**Conclusion:**

We describe the development of a new in vitro model for HIV-1 latency and discuss the advantages of this system. The data suggest that HIV-1 proviral latency is not restricted to resting T cells, but rather an intrinsic property of the virus.

## Background

With the introduction of highly active antiretroviral therapy (HAART) against HIV-1 it has become possible to stably reduce the viral load below the detection limit in most patients (reviewed in [1]). Current therapies target various steps of the virus replication cycle but can only prevent new infections, without an impact on already infected cells or the integrated provirus. Clearance of infected cells is possible only by cell death or recognition by the host immune system. Consequently, HIV-1 can persist in long-lived reservoirs of different cell types. These cellular reservoirs may differ in the magnitude of viral latency, ranging from low-level virus production that does not trigger immune recognition to truly latent proviruses [2-7]. Viral infection of resting and quiescent cells can lead to a pre-integration complex that fails either to complete reverse transcription or to integrate. These complexes are stable for only a few days and are therefore not important for long term latency [8-11]. Another component of the reservoir consists of productively infected lymphocytes (CD4^+ ^T cells) that reverted from an activated state to a quiescent state as a consequence of establishing immunological memory [2]. In the resting state, these cells fail to produce virus, but these proviruses can be reactivated later on [8-12]. These resting T cells persist with an average half-life of 44 months [13], and it can be estimated that it will take more than 60 years to eradicate this reservoir [14]. Numerous attempts have been made to activate these latently infected cells in order to expose these viral reservoirs to antiviral drugs, thus far with limited success in patients [2].

A key determinant for viral transcription after proviral DNA integration is the status of cellular transcription factors that activate the basal LTR promoter to express the viral Tat protein that induces an autoregulatory expression loop. Tat binds to the TAR RNA hairpin encoded by the LTR and recruits the cyclin T1-CDK9 complex to enhance viral transcription [15-18]. The HIV-1 subtypes each encode a unique set of transcription factor binding sites in their LTR, suggesting the intriguing possibility that the subtypes differ in their latency profile [19,20]. In addition it has been demonstrated that unfavorable proviral integration sites can also result in low level basal transcription and cause latency [21,22].

An important obstacle in latency research is the lack of a good experimental model system. Patient samples are not well suited for analysis since latently infected cells can hardly be distinguished phenotypically from uninfected cells. The presence of proviral DNA is an obvious difference, but there are currently no validated methods to quantify or select these cells. It is also difficult to distinguish cells with defective versus latent proviruses, although reactivation is an option [2,23]. In vitro studies frequently use the latently infected cell lines U1 and ACH-2, although latency is caused by mutational disruption of the regular Tat-TAR axis [2,24,25]. As an alternative, J-Lat cells were selected for lack of GFP expression from a proviral construct, which could be activated by TNFα treatment [21].

In this study, we used the doxycycline (dox) dependent HIV-rtTA variant [26-28] to generate a new HIV-1 latency model. We constructed stable cell lines with a HIV-rtTA provirus that is completely dependent on dox for virus production. This inducible, fully replication competent virus forms an ideal tool to study certain aspects of proviral latency. With wild-type HIV-1, one cannot stop ongoing virus replication that eventually kills the host cell. With HIV-rtTA, it is possible to freeze the proviral state by dox withdrawal and subsequent reactivation can be done at will by simple dox addition. Surprisingly, we measured a high degree of HIV-rtTA proviral silencing in the actively dividing T cell line SupT1. With clone-to-clone variation, only 0.1–10% of the provirus containing cells can be induced to express virus. Reactivation of these proviruses was possible by activation of the NF-κB pathway and by influencing genomic DNA methylation. Follow-up experiments in several other T cell lines and with wild-type HIV-1 support these findings. These results indicate that HIV-1 latency is established frequently in actively dividing cells.

## Results

### Strong silencing of HIV-rtTA in SupT1 T cells

**Figure 1:**
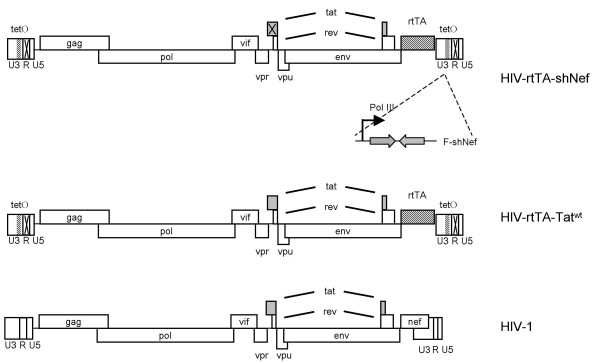
**Schematic overview of HIV-1 variants used in this study**. In the HIV-rtTA variant, the rtTA gene is inserted in place of the nef gene and TetO binding sites are inserted in the HIV-1 promoter. Inactivation of the Tat-TAR axis is obtained by mutations in the R region and the Tat protein. The shNef expression cassette is inserted in the U3 region. The Tat inactivating mutation (indicted by X) in HIV-rtTA has been restored in the HIV-rtTA Tat^wt ^variant.

We used the dox-dependent HIV-rtTA virus to study HIV-1 latency (Fig. 1)[27]. In this conditional live virus, the normal Tat-TAR transcriptional axis has been replaced by components of the Tet-On system, but the regular HIV-1 promoter elements are conserved [29,30]. Infection of cells in the absence of dox will allow the establishment of a transcriptionally silent provirus as a new model for HIV-1 latency. Subsequent dox-addition will activate the provirus on command. We recently used this virus to construct the HIV-rtTA-shNef variant with a polymerase III (Pol-III) driven shRNA expression cassette that targets the wild-type HIV-1 *nef *gene, which is lacking in this construct (Fig. 1). This shRNA is produced constitutively whereas the viral LTR promoter is under dox-control.

Consequently, the shNef-producing cells with this inducible provirus should be protected against wild-type HIV-1 infection. We produced 24 cell clones derived from the SupT1 T cell line upon HIV-rtTA-shNef infection and demonstrated resistance to subsequent HIV-1 challenge in most clones (Table 1, third column). However, five of the 24 cell clones remained sensitive to HIV-1 superinfection and further analysis indicated that transcriptional silencing of the Pol-III cassette may have occurred [31].

**Table 1:** Overview of SupT1 clones with integrated HIV-rtTA provirus

Clone	HIV-rtTA gDNA PCR	HIV-1 resistant^1^	Dox-induced				Number detected proviruses
				
			HIV-rtTA replication^2^	Intracellular CA-p24^3^			
				(24 h)	(48 h)	M.F.I.^5^	
B5	+	-	-	0.01	0.04	-	-^4^
B8	+	-	-	0.38	0.34	62	-
C2	+	+	-	0.04	0.00	-	1
C4	+	+	-	0.01	0.00	-	-
C7	+	+	-	0.00	0.00	-	2
D2	+	+	-	0.01	0.06	-	1
D10	+	-	-	0.01	0.02	-	-
D11	+	+	-	0.02	0.01	-	1
E4	+	+	-	0.00	0.01	-	2
F3	+	-	-	0.00	0.01	-	-
H7	+	+	-	0.00	0.00	-	3
							
B4	+	+	+	0.38	0.50	70	1
B6	+	+	+	0.22	0.19	78	5
B9	+	+	+	1.34	1.66	69	3
C5	+	+	+	0.72	0.82	106	3
C11	+	+	+	0.01	0.04	-	3
D4	+	+	+	0.07	0.02	-	4
D6	+	+	+	0.07	0.02	-	1
E7	+	+	+	0.43	0.59	100	-
E11	+	+	+	0.05	0.02	-	-
F7	+	+	+	10.52	11.54	49	2
F8	+	+	+	1.31	2.16	82	1
F9	+	-	+	0.07	0.02	-	1
G2	+	+	+	0.02	0.01	-	1

^1 ^Wild-type HIV-1 LAI superinfection

^2 ^HIV-rtTA replication scored by syncytia formation and extracellular CA-p24 production

^3 ^Percentage CA-p24 positive cells

^4 ^-; not detected

^5 ^M.F.I. is the mean fluorescence intensity of the CA-p24 positive cells (48 h dox)

To expand on this putative silencing effect we analyzed HIV-rtTA virus production and replication in all 24 clones. An HIV-1 specific PCR on genomic DNA confirmed that all cell lines have at least one provirus (Table 1, second column). Prolonged culturing of these clones with dox demonstrated that 13 clones produce replication-competent HIV-rtTA virus, but 11 clones are unable to initiate a spreading virus infection. The positive clones are grouped in the bottom part of Table 1. The percentage of cells with a dox-inducible provirus was analyzed by intracellular staining for the CA-p24 protein after 24 h and 48 h dox-induction (Table 1). To avoid multiple rounds of infection, we added the potent fusion inhibitor T1249. Surprisingly, only few cells of the clonal cultures responded to dox addition, ranging from 0% to approximately 10% of the cells in clone F7 (Table 1). In general, a correlation between CA-p24 production and HIV-rtTA replication was observed, with few exceptions. The clones that do not support dox-induced virus replication also lack any detectable CA-p24 production, with the exception of clone B8. An obvious explanation of this phenomenon is the presence of a defective provirus that would prevent virus production, although a frequency of 11 out of 24 clones seems very high. Most importantly, among the 13 clones that do contain a viable and replication-competent provirus, only a minority of the cells (0–10%) is able to produce virus after dox administration, suggesting massive silencing of the HIV-rtTA-shNef provirus in the SupT1 T cell line.

**Figure 2:**
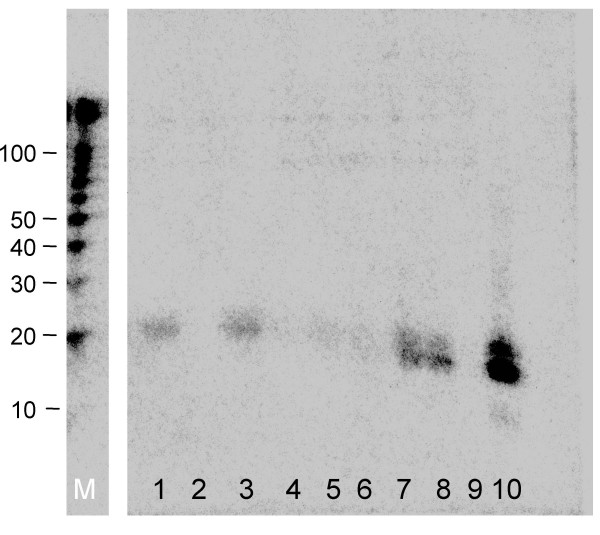
**Northern blot analysis of shRNA production in rtTA-shNef SupT1 cell lines**. RNAs from the different HIV-rtTA-shNef SupT1 clones were isolated and separated on an agarose gel. After blotting the membrane was probed with a LNA probe against the shNef. Lane M, marker; lane 1, shNef clone B9; lane 2, clone C10 (a negative control cell line without a provirus); lane 3, shNef clone D2; lane 4, shNef clone D11; lane 5, shNef clone F7; lane 6, shNef clone F8; lane 7 and 8 contain positive controls from a transfection with the F-shNef^- ^and F-shNef^+ ^plasmid [31]; lane 9 contains a negative empty-vector control; lane 10 in vitro produced shNef RNA.

We screened several of the HIV-resistant cell clones for shRNA expression by Northern blot analysis (Fig. 2). We observed a quite variable expression level among the clones, indicating that a relative low shRNA level is sufficient to block wild-type virus replication. There was no significant correlation between the level of Pol-III driven shRNA expression and the level of Pol-II driven CA-p24 production. We thusfar only discussed the percentage of cells that start producing CA-p24 upon dox-addition as a measure of proviral latency. In fact, we also scored the absolute level of CA-p24 production in positive cells from the different clones, but measured very similar expression levels (Table 1). Interestingly, this rather uniform CA-p24 expression level in HIV-producing cells from different clones indicates that the intrinsic transcriptional activity is determined by the provirus itself and relatively independent of the integration locus. This makes sense as it may be important for the virus to reach a precisely fine-tuned level of gene expression for optimal virus production. Consistent with this idea, we observed promoter modulation in virus evolution experiments [32].

### Activation of latent HIV-rtTA provirus

**Figure 3:**
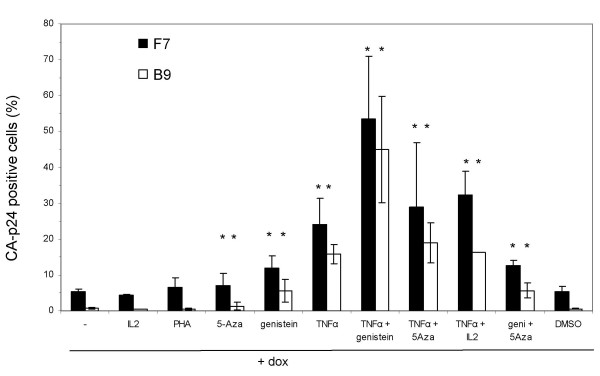
**Silencing and reactivation in HIV-rtTA SupT1 cell lines**. The HIV-rtTA SupT1 cell lines F7 and B9 were induced with dox or with dox in combination with the indicated activators. DMSO is the solvent for genistein and therefore used as an additional control. Statistical significance was determined with a two-tailed student's T test for each combination versus dox alone (GraphPad Prism). Significant changes (*P*-value < 0.05) are indicated with asterisks.

To analyze if dox-induction of the integrated HIV-rtTA provirus is prohibited by silencing/latency of the HIV LTR promoter we tried to activate the provirus in these cells. We tested regular T cell activators, demethylation inducers and cell cycle blockers. TNFα induces activation of the NF-κB pathway, which plays an important role in reactivation and preventing HIV-1 latency [2,14,21,33,34]. 5-azacytidine (5-Aza) causes demethylation of genomic DNA and can overcome latency. Genistein induces a G2 arrest, stimulates lentiviral gene transfer [35] and HIV-1 replication [36]. These compounds were tested individually and in combination. We determined the percentage of CA-p24 positive cells after 24 h dox-induction with or without these effectors. This analysis was performed on the high-producer SupT1 cell clone F7 and the low-producer clone B9. TNFα increases the percentage of CA-p24 producing cells upon dox-induction 5-fold for the clone F7 and more than 24-fold for clone B9, thus boosting the percentage of positive cells in both cases to approximately 20% (Fig 3). In other words, the impact of activation is more pronounced in the restricted cell clone B9 compared to the high-producer clone F7. Genistein and 5-Aza also partially resolve the proviral latency, suggesting that expression from a HIV-rtTA provirus can be triggered through multiple routes. Regular T cell activators like IL2 and PHA do not increase the fraction of HIV-rtTA producing cells in response to dox. A particular potent combination to avoid latency is TNFα with genistein, yielding approximately 50% positive cells for both the F7 and B9 clones. The effectors genistein, 5-Aza and TNFα also seem to have a positive effect on basal gene expression from the HIV-rtTA provirus without dox, but the low percentage of CA-p24 positive cells (≤ 0.01%, results not shown) does not allow accurate measurements.

The ability to activate a provirus is likely related to the specific integration site and the number of proviruses. The integration sites in the different clones were identified by linker mediated PCR [37] and/or inside-out PCR [38]. Despite the use of two different techniques, it was not possible to identify the actual integration sites for all clones. Thus, the number of 1 to 5 integration sites in the clones should be regarded as a minimal estimate. The presence of multiple proviruses will make the cell less likely to exhibit the latency phenotype. No strict correlation was apparent between dox inducibility and the number of HIV-1 proviruses, but inspection of the data in Table 1 suggests a trend towards more proviruses in the group that allows HIV-rtTA replication (gray panel in the bottom part of Table 1). On average, the number of detected proviruses in this group is 1.9 (range 0–5) versus 0.9 (range 0–3) in the non-producer cell clones. We also analyzed specific characteristics of the integration sites (chromosome number, gene density, intra- versus inter-genic, proximity to transcription start sites, proximity to CpG Islands) but did not notice any striking trend between high- versus low-CA-p24 producing cell clones.

### HIV-rtTA with an active Tat protein also demonstrates latency

The latency phenotype in SupT1 cells may be a specific property of the HIV-rtTA variant virus. For instance, this virus variant encodes a mutant Tat protein (Tyr26Ala) that is unable to support Tat-mediated transcription [39]. To test this possibility, we also generated SupT1 clones with an integrated HIV-rtTA variant that encodes a wild-type Tat protein. We found that all clones exhibit a similarly low percentage of CA-p24 producing cells upon dox-induction as observed for the Tat-minus HIV-rtTA variants (results not shown). Thus, a wild-type Tat protein cannot prevent the establishment of proviral latency, although one should realize that Tat production will be minute in the absence of dox. Alternatively, during selection of the clones, there is no transcription from the HIV-rtTA promoter and this could theoretically result in active silencing of the provirus and subsequently block dox activation. However, we observed the same phenomenon when the HIV-rtTA or HIV-rtTA Tat^wt ^clones were generated in the absence or continuous presence of a low level of dox (results not shown).

### Latency effects of wild-type HIV-1 in SupT1 cells

**Figure 4:**
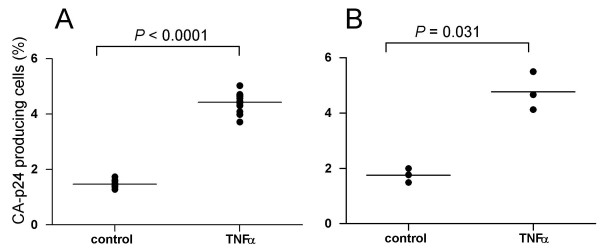
**Latent HIV-1 infection in SupT1 T cells**. TNFα-induced reactivation of silent HIV-1 proviruses in SupT1 cells was analyzed by determining the percentage of CA-p24 producing cells by intracellular FACS analysis. (A) After a single round infection with the LAI isolate, the culture was split and one culture activated for 24 h with TNFα and the other used as a control. (B) The control culture was maintained for one week and split again into a TNFα treated and control culture. Statistical significance was determined with a two-tailed student's T test (GraphPad Prism).

To establish if the observed latency is also seen for wild-type HIV-1, we set up a single round infection system with the HIV-1 LAI isolate in the SupT1 cell line. Multiple rounds of infection were prevented by addition of T1249 at 2 h after infection. After 24 h, the infected culture was split in two and TNFα was added to one culture. Intracellular CA-p24 staining was performed after another 24 h. We measured a 3-fold increase of the percentage of virus producing cells in the presence of TNFα, indicating profound silencing without activation of the T cell line (Fig. 4A). We should point out that this experiment is more difficult to interpret than the previous HIV-rtTA analyses. For instance, it is possible that TNFα addition has other stimulatory effects on the infection process, e.g. reverse transcription or integration rather than on reducing latency. To exclude these possibilities, the TNFα induction was repeated with the control culture 7 days after the initial infection. Again, TNFα increased the percentage of virus producing cells 2- to 3-fold (Fig. 4B). The results indicate that infection of SupT1 cells with wild-type HIV-1 frequently results in the establishment of a latent provirus.

**Figure 5:**
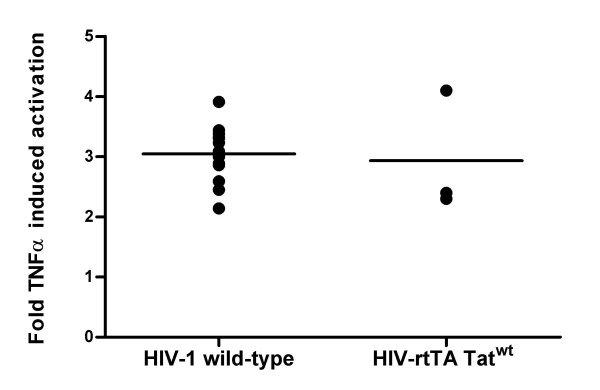
**Latent HIV-1 and HIV-rtTA infection in SupT1 T cells**. TNFα-induced reactivation of silent HIV-1 proviruses in SupT1 cells was analyzed by determining the percentage of CA-p24 producing cells by intracellular FACS analysis. After a single round infection with HIV-1 (LAI isolate) or the HIV-rtTA-Tat^wt^, each culture was split and either activated for 24 h with TNFα or not. The fold TNFα activation is the percentage CA-p24 positive cells in the culture with TNFα divided by the percentage of CA-p24 positive cells in the control culture.

In the single-round infection experiments with wild-type HIV-1, it is not easy to determine the frequency of latent versus active proviruses. With a low multiplicity of infection, only a small percentage of the cells will be infected and this results in unreliable FACS data. With a higher multiplicity of infection, cells are likely to contain multiple proviruses and a single active provirus will dominantly mask multiple silent proviruses. With the cell clone experiments that are possible with the HIV-rtTA virus, we found a very high frequency of latent proviruses. To directly compare latency properties of the wild-type and HIV-rtTA virus, the single round infection experiment was repeated with both viruses. A similar increase in the percentage of virus producing cells was observed upon TNFα addition (Fig. 5), indicating similar latency frequencies.

**Figure 6:**
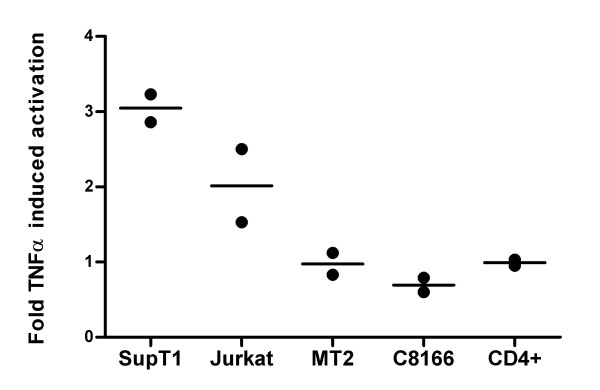
**Latent HIV-1 infection in various T cell lines**. TNFα-induced reactivation of silent HIV-1 proviruses in the indicated T cell lines was done as described in Figure 4. The fold TNFα activation is the percentage CA-p24 positive cells in the culture with TNFα divided by the percentage of CA-p24 positive cells in the control culture.

To broaden the analysis of this latency phenotype, we used other T cell lines in single round infections with the wild-type HIV-1 LAI isolate. TNFα also increased the percentage of CA-p24 positive Jurkat cells, but no such effects were observed in the HTLV-1 transformed T cell lines C8166 and MT2, and in PHA/IL2 activated CD4^+ ^T lymphocytes (Fig. 6). These results indicate that TNFα mediated latency suppression might be cell type specific.

## Discussion

We previously constructed a unique HIV-1 variant in which the Tat-TAR transcription motifs were inactivated and replaced by the inducible Tet-On system [27]. In this study, this HIV-rtTA virus was used to generate cell lines with a dox-controllable HIV provirus. These cell lines harbor a silent provirus that is completely dependent on dox addition for activation but that contains the original transcription factor binding sites in the HIV-1 LTR promoter for regulated transcription. An additional important advantage of this system is that no selection was used to obtain these cell lines, thus avoiding any bias towards certain activation markers. These clonal cell lines are therefore an ideal tool to study HIV-1 transcriptional latency. We screened these cell lines for dox-induced intracellular CA-p24 production by FACS analysis. This allows us to discriminate at the cellular level between active and silent proviruses.

A surprising observation was that, although experiments were performed in the actively dividing T cell line SupT1, only 0.1–10% of the cells is able to produce CA-p24 after dox addition. These results indicate that HIV-1 latency is not restricted to non-dividing cells, but apparently also frequently occurs in actively dividing cells. In fact, it seems that the establishment of proviral latency is the default pathway in the SupT1 T cell line. The results with the Jurkat T cell line confirm this idea. It could be argued that silencing is induced because our HIV-rtTA variant will be transcriptionally silent as long as we do not provide dox. However, no difference in viral infectivity was scored between single round infections in which dox was continuously present or added at later time points (17 h, 41 h). We do not think that this observation is unique for the dox-inducible HIV-rtTA virus since we also measured a similar phenomenon for the wild-type HIV-1 LAI isolate in these SupT1 cells. In addition, it has been demonstrated that HIV-1 uses the cellular miRNA machinery to maintain proviral latency in resting cells [40] and it has been shown that the NF-κB sites in the LTR promoter can be used for maintenance of latency [41,42].

Analysis of the integration sites of HIV-rtTA in these cell lines showed only a weak positive correlation between the number of integration sites and the ability to response to dox addition. We also did not observe striking correlations between dox response and local genomic DNA features around the integration site, but a significantly larger sample number is required to identify such correlations.

The latent provirus could be activated by TNFα, a commonly used reagent to reduce HIV-1 latency, and by genistein and 5-Aza that alter DNA methylation. Combinations of these reagents further increased the level of provirus activation, thus indicating an additive effect. In contrast, the regular T cell activators PMA and IL2, which can be used for HIV-1 reactivation in resting cells, have no (additional) effects in these SupT1 cell lines. Huang et al. recently demonstrated that miRNAs contribute to latency in resting cells and that activation of the cells by PMA and IL2 reactivates transcription [40]. This activation does not occur in SupT1 cells, in agreement with their activated state. These combined results suggest the presence of different cellular reservoirs for HIV-1 latency. Each reservoir may require a specific activation strategy. This means that therapy that aims to purge these latent viruses will be more difficult, and this could explain why attempts thus far have been unsuccessful.

To confirm these latency properties for wild-type HIV-1, we set up a single cycle infection assay and analyzed proviral activity upon TNFα addition. Despite this more indirect analysis, TNFα increased the percentage of virus-producing cells 2 to 3 fold in infections with wild-type HIV-1 (LAI isolate) in SupT1 and Jurkat cells. These results are in agreement with another study [43] that described strong latency effects with HIV-1 based lentiviral vectors in Jurkat cells and with J-lat cell lines that were generated by sorting for Jurkat cells with a latent HIV-1 provirus that can be activated by TNFα [21].

We did not observe any TNFα stimulation in PHA/IL2 activated CD4+ cells and the HTLV-1 infected cell lines C8166 and MT2. TNFα stimulates proteolytic degradation of IkB by the proteasome [44-46]. This degradation liberates NF-kB and allows its nuclear translocation and subsequent activation of the HIV-1 LTR. TNFα mediated transcriptional activation of HIV-1 and LTR-driven reporter constructs has been described in SupT1 [20], Jurkat [25], and HeLa cells [47]. TNFα can also activate the latent HIV-1 cell lines U1 (based on the promonocyte U937 cell line) and ACH2 cells (based on A3.01) [24,48]. In these cell lines, NF-kB driven expression can overcome the defective Tat-TAR axis present in these proviruses. The absence of a TNFα effects in PBMC and in the MT2/C8166 cell lines indicates that it is specific for certain T cell lines, possibly related to their differentiation stage. For instance, the SupT1 cell line represents an early stage in T cell development and these CD4^+ ^CD8^+ ^cells are relative rare in PBMC, and even absent in our CD8^+^-depleted CD4^+ ^preparations. Alternatively, a high level of endogenous NF-kB or activation of the NF-kB pathway by endogenous TNFα production by monocytes, which are still present in the T cells preparations [49], could explain the lack of a TNFα effect in these assays. In case of the HTLV-1 transformed cell lines, the absence of a TNFα response could also be the result of expression of the viral Tax trans-activator protein, which blocks TNFα activation [50-55].

In HIV-1 infected individuals the number of integrated HIV-1 DNA copies is generally 100-fold higher than the number of cells that produce replication-competent virus after activation (reviewed in [2]). This discrepancy is usually explained by the prevalence of defective proviral DNA genomes due to the high error rate of the HIV-1 RT enzyme and/or activity of gene-modifying enzymes like APOBEC3G. We could not demonstrate any TNFα effect in PBMC, but demonstrated with the HIV-rtTA clones that TNFα stimulation is not sufficient to completely prevent the establishment of latency. This could mean that latency also occurs in activated primary cells, providing an alternative explanation for the high number of integrated DNA copies. Furthermore, the generation of the latent reservoir in resting memory cells is thought to be mainly the result of reversion of an infected cell from an active state to a resting state. This means that these cells have to survive HIV-1 replication and escape from immune recognition long enough to establish post-integration latency. It seems likely that this reversion is much easier for HIV-infected cells that have a latent provirus.

A high frequency of latently infected cells has important consequences for our understanding of HIV-1 persistence, especially when present activated T cells. For instance, this means that far more infected cells will escape from immune surveillance then previously anticipated and these cells will also be unresponsive to any drug treatment. Productive infection of activated T cells results in reduced susceptibility to new infections due to downregulation of CD4 and cell cycle arrest that is imposed by viral proteins. Activated cells with a latent provirus do not express viral proteins and these cells can therefore be re-infected with the same efficiency as uninfected cells. This makes superinfection a regular event, which is in agreement with recent findings [56,57]. Productive superinfection will likely reactivate the already present latent proviral DNA and result in cells that produce two (or even more) virus strains. Consequently, recombination can occur at a high frequency. The proviral copies in these cells have been established at different times, and this allows the mixing of historical and more recent virus sequences.

In conclusion, we have developed a new HIV-1 latency model system that uses the dox inducible HIV-rtTA virus variant. In combination with FACS analysis, this system allows the quantitative analysis of transcription activation of HIV-1 from a latent provirus. The HIV-rtTA virus is ideally suited to study the molecular details of the integrated HIV-LTR promoter in an active and latent state. The system can also be used to quickly quantify the effects of reactivation protocols. Unlike other latency cell systems that were selected for specific reactivation properties (e.g. TNFα responsiveness in the J-Lat system), our cell clones are obtained without any selection. Our data indicate that HIV-1 proviral latency is not restricted to resting cells, but also apparent in actively dividing cells. In cell culture clones, we measured a dramatically differential response of individual cells to dox induction. These observations are in agreement with a stochastic model of HIV-1 gene expression that was proposed by Weinberger [58,59]. This model, based upon a simplified lentiviral vector system with the Tat-TAR transactivation feedback loop, demonstrates that stochastic, random fluctuations in the Tat protein level determine if LTR-mediated transcription will initiate. We observed similar fluctuations with our replication competent virus system in which the Tat/TAR feedback loop is replaced with an inducible rtTA feedback loop.

## Methods

### Cells and viruses

C33A cervix carcinoma cells (ATCC HTB31) [60] were grown as a monolayer in advanced Dulbecco s minimal essential medium supplemented with 1% (v/v) fetal calf serum (FCS), 40 U/mL penicillin, 40 μg/mL streptomycin, 20 mM glucose and minimal essential medium nonessential amino acids at 37°C and 5% CO_2_. The cells were transfected with plasmid DNA of the HIV-1 LAI molecular clone [61] and doxycycline (dox)-dependent HIV-rtTA derivatives [26,31,62] by the calcium phosphate method as described previously [63]. For analysis of the percentage of dox-inducible cells, new rounds of infection were prevented by addition of the entry inhibitor T1249 [64].

The human T lymphocytic cell lines SupT1 (ATCC CRL-1942) [65], MT2, C8166 and Jurkat (ATCC TIB-152) were cultured in advanced RPMI 1640 medium (Gibco BRL, Gaithersburg, MD) supplemented with 1% (v/v) FCS, 40 U/mL penicillin, and 40 μg/mL streptomycin at 37°C and 5% CO_2_. HIV-1 infections were performed with C33A-produced virus stocks of the HIV-1 LAI molecular clone [61].

SupT1 cells transduced with various HIV-rtTA proviruses (without dox) were used for limiting dilution to obtain cell clones. Serial dilutions (3-fold) were prepared in 96-well plates. Fresh medium was added every 10 days. As an additional control for the presence of the provirus in all cells, we performed an additional round of limiting dilution with the HIV-rtTA-shNef clones D2, B9 and F7. All tested subclones (11 for D2, 6 for B9 and 15 for F7) were positive in a genomic DNA PCR for the provirus (Table 2). The frequency of dox-induction was similar to the parental cell line, although most of the F7 derived subclones showed relative low induction levels, and only a few F7 subclones showed very high induction (Table 2).

**Table 2:** Characteristics of second round limiting dilution of shNef clones D2, B9 and F7

Clone	HIV-rtTA gDNA PCR	Intracellular CA-p24 (24 h)
D2-1B	+	0.10
D2-2C	+	0.02
D2-4A	+	0.01
D2-4B	+	0.07
D2-5D	+	0.04
D2-6D	+	0.01
D2-6G	+	0.04
D2-7F	+	0.02
D2-8C	+	0.04
D2-10D	+	0.01
D2-12G	+	0.01
		
B9-2F	+	2.07
B9-2G	+	0.12
B9-3H	+	0.51
B9-4E	+	0.71
B9-5F	+	0.13
B9-11G	+	0.29
		
F7-2B	+	0.41
F7-3F	+	0.2
F7-3G	+	0.74
F7-4C	+	0.22
F7-5F	+	0.35
F7-5H	+	0.11
F7-6B	+	1.41
F7-6D	+	69.05
F7-7G	+	0.18
F7-8F	+	14.30
F7-9D	+	1.25
F7-10A	+	0.11
F7-10E	+	15.75
F7-10F	+	0.28
F7-10G	+	0.41

Peripheral blood mononuclear cells (PBMC) were isolated from different healthy donors, and each batch consists of a mixture of four different donors. PBMC were cultured as the T cell lines cells, but with the addition of 100 U/mL human IL2 after initial PHA (5 μg/mL) stimulation for 2 days. CD4^+ ^cells were isolated by depletion of CD8^+ ^cells with Dynabeads^® ^M450 CD8 (Dynal Biotech ASA, Norway) according to the supplier s instructions.

### Chemicals

Genistein (Sigma G6649) was prepared as 1000× stock solution (30 mM) in DMSO. 5-Azacytidine (Sigma A1287) was prepared as 50× stock solution (500 μM) in advanced RPMI 1640. TNFα (Invitrogen PHC3015) was prepared as 2000× stock solution (10 μg/mL) in sterile milliQ H_2_O. T1249 (WQEWEQKITALLEQAQIQQEKNEYELQKLDKWASLWEWF, Pepscan Therapeutics BV, Lelystad, The Netherlands) was obtained as 10.000× stock solution. Doxycycline (Sigma D9891) was prepared as a 1000× stock in sterile milliQ water and used at a final concentration of 1000 ng/mL.

### DNA and RNA manipulations

Plasmid preparations were performed using Qiagen isolation kits. A Northern analysis of shRNA was performed as described previously [31]. Approximately 15 × 10^6 ^cells of the different HIV-rtTA-shNef SupT1 clones were lysed and RNAs were isolated using the mirVana™ miRNA isolation kit (Ambion). Gel electrophoresis of 7 μg RNA (3.5 μg RNA for the transfected controls) was performed on a 15% acrylamide Novex^® ^TBE-Urea gel (Invitrogen) at 180 V in 1×TBE buffer (90 mM Tris, 90 mM boric acid and 2 mM EDTA, pH 8.3). RNA was transferred onto a positively charged nylon membrane (Boehringer Mannheim) for 2 h at 80 V and cross-linked to the membrane with a UV crosslinker (Stratagene). A 19-nt LNA-molecule (Eurogentec) with a sequence similar to the shRNANef target sequence (5' GTGCCTGGCTAGAAGCACA 3' ; locked nucleotides are underlined) was used as a probe. The probe was 5' end labeled using the kinaseMax kit (Ambion) in the presence of 2 μL of [-32P]ATP (0.37 MBq/μL, Amersham Biosciences) and purified over a MicroSpin™ G-25 column (GE Healthcare). Prehybridization and hybridization was done in ULTRAhyb buffer (Ambion) at 42°C for 30 min and 18 h, respectively. The membrane was washed twice at 42°C with low-stringency buffer (2×SSC, 0.1% SDS). Images were obtained using the Typhoon Trio phosphorimager (GE Healthcare).

### CA-p24 intracellular staining and fluorescence-activated cell sorting

Flow cytometry was performed with RD1 or FITC-conjugated mouse monoclonal anti-CA-p24 (clone KC57, Coulter). Cells from a 1 mL culture sample were collected (4 min 4000 rpm, Eppendorf centrifuge) and fixated in 250 μL 4% formaldehyde for 5 min at room temperature. The cells were washed with 500-μL BD Perm/Wash™ buffer (BD Pharmingen) and stained for at least 30 min at 4°C in 20 μL of BD Perm/Wash™ buffer and 5 μL of the appropriate antibody (diluted 1 in 100). Excess antibody was removed by washing the cells with 500-μL BD Perm/Wash™ buffer. The cells were collected and resuspended in 750 μL FACS buffer (PBS with 2% FCS). Cells were analyzed on a FACScalibur flow cytometer with CellQuest Pro software (BD biosciences, San Jose, CA). Cell populations were defined based on forward/sideward scattering. Isotype controls (clone MsIgG-RD1, Coulter) or uninduced control cells (minus dox) were used to set markers. The data from different assays was corrected for between-session variation with the factor correction program [66].

### CA-p24 Elisa

Culture supernatant was heat inactivated at 56°C for 30 min in the presence of 0.05% Empigen-BB (Calbiochem, La Jolla, USA). CA-p24 concentration was determined by a twin-site ELISA with D7320 (Biochrom, Berlin, Germany) as the capture antibody and alkaline phosphatase-conjugated anti-p24 monoclonal antibody (EH12-AP) as the detection antibody. Detection was done with the lumiphos plus system (Lumigen, Michigan, USA) in a LUMIstar Galaxy (BMG labtechnologies, Offenburg, Germany) luminescence reader. Recombinant CA-p24 produced in a baculovirus system was used as the reference standard.

### Determination of proviral integration sites

HIV-1 integration sites were determined by two methods: linker-mediated PCR (LM-PCR) as described [37] and inside-out PCR (IO-PCR) with minor adaptations on the original protocol [38]. Briefly, genomic DNA was isolated with the DNeasy kit (Qiagen). For LM-PCR, 0.5 μg genomic DNA was cut with *Sac*I and *Tru*9I and ligated with primers RJ037 (5' GTA ATA CGA CTC ACT ATA GGG CTC CGC TTA AGG GAC 3') and RJ038 (5' PO_4_-TAG TCC CTT AAG CGG AG-NH_2 _c_6 _3'). Subsequently, the mixture was used as template in a PCR reaction with primers RJ039 (5' AGT GCT TCA AGT AGT GTG TGC C 3') and RJ041 (5' GTA ATA CGA CTC ACT ATA GGG C 3'), followed by a nested PCR with primers RJ040 (5' GTC TGT TGT GTG ACT CTG GTA AC 3') and RJ042 (5' AGG GCT CCG CTT AAG GGA C 3'). All PCR reactions were set up with 2× reddye™ premix (Thermo Fisher Scientific, Waltham, USA).

For the inside-out PCR, we digested 25 ng genomic DNA with *Pst*I, *Nsi*I or both enzymes and allowed self-ligation after inactivation of the restriction enzymes for 20 min at 80°C by addition of T4 ligase. This ligation mix was used as template in a PCR reaction with primers A1095 (5' GGA CAT CAA GCA GCC ATG CAA AT 3') and A2623 (5' TAT GCA GCA TCT GAG GGC TC 3') for *Pst*I digestions or A2790 (5' GGA TAG AGA TAA AAG ACA CC 3') and A2623 for the other enzyme combinations, followed by a nested PCR with primers A1096 (5' AAA GAG ACC ATC AAT GAG GAA GCT 3') and A2624 (5' GCA GTT CTT GAA GTA CTC CG 3') or primers A2596 (5' CAT CAGC CCA TAT CAC CTA GAA CT 3') and A2624, respectively.

Nested PCR products were analyzed by agarose gel electrophoresis and sequenced directly in case a single band was observed. Otherwise, the PCR products were first cloned into a Topo vector (Invitrogen) according to the suppliers instructions and individual clones with an insert were sequenced [28]. The obtained sequences were trimmed for primers and HIV-1 sequences and compared to the human genome using human BLAT search [67] on the UCSC website [68] and BLAST on the NCBI server [69].

### Single round infection assays

SupT1 cells were infected with HIV-1 virus stocks of the primary CXCR4-using LAI isolate. After two hours, excess virus was washed away and the cells were further cultured in the presence of entry inhibitor T1249 to block subsequent rounds of viral entry. The culture was split after 24 h post-infection, and TNFα was added to one culture. After another 24 h and 48 h, we measured extracellular CA-p24 production by Elisa in the cell culture medium and intracellular CA-p24 by FACS analysis.

## Competing interests

The authors declare no potential conflict of financial or personal interest related to the results communicated in this paper.

## Authors' contributions

REJ was responsible for designing, performing and writing the manuscript. EMW was responsible for the northern blot analysis and constructed the shNef clones. MLvG contributed to the flow cytometry experiments. BB was responsible for design and writing of the manuscript. All authors read and approved the final manuscript.
